# Relationship between Spectral Characteristics of Spontaneous Postural Sway and Motion Sickness Susceptibility

**DOI:** 10.1371/journal.pone.0144466

**Published:** 2015-12-14

**Authors:** Rafael Laboissière, Jean-Charles Letievant, Eugen Ionescu, Pierre-Alain Barraud, Michel Mazzuca, Corinne Cian

**Affiliations:** 1 CNRS, LPNC, F-38000 Grenoble, France; 2 Univ. Grenoble Alpes, LPNC, F-38000 Grenoble, France; 3 INSERM/CNRS, CRNL, F-69675 Bron, France; 4 Service Explorations Audio-Vestibulaires, HCL, F-69003 Lyon, France; 5 CNRS, TIMC-IMAG, F-38000 Grenoble, France; 6 Institut de Recherche Biomédicale des Armées, F-91223 Brétigny-sur-Orge, France; Tokai University, JAPAN

## Abstract

Motion sickness (MS) usually occurs for a narrow band of frequencies of the imposed oscillation. It happens that this frequency band is close to that which are spontaneously produced by postural sway during natural stance. This study examined the relationship between reported susceptibility to motion sickness and postural control. The hypothesis is that the level of MS can be inferred from the shape of the Power Spectral Density (PSD) profile of spontaneous sway, as measured by the displacement of the center of mass during stationary, upright stance. In Experiment 1, postural fluctuations while standing quietly were related to MS history for inertial motion. In Experiment 2, postural stability measures registered before the onset of a visual roll movement were related to MS symptoms following the visual stimulation. Study of spectral characteristics in postural control showed differences in the distribution of energy along the power spectrum of the antero-posterior sway signal. Participants with MS history provoked by exposure to inertial motion showed a stronger contribution of the high frequency components of the sway signal. When MS was visually triggered, sick participants showed more postural sway in the low frequency range. The results suggest that subject-specific PSD details may be a predictor of the MS level. Furthermore, the analysis of the sway frequency spectrum provided insight into the intersubject differences in the use of postural control subsystems. The relationship observed between MS susceptibility and spontaneous posture is discussed in terms of postural sensory weighting and in relation to the nature of the provocative stimulus.

## Introduction

Motion sickness (MS) is a general term for the adverse signs that are provoked by exposure to certain types of real or apparent motion [1, 2]. The sensory conflict or neural mismatch hypothesis of MS is widely accepted [3–5]. According to this hypothesis, MS is considered as the result of a conflict between the information processed within a multimodal sensory system, whose function is to determine the individual’s motion relative to the environment. A conflict occurs when the integrated sensory signal is compared and found at variance with previously recognized and stored motion paradigms. This results in a mismatch signal that initiates MS symptomatology. Some authors have claimed that the only conflict of interest is the one related to the internal representation of the vertical direction [6, 7]. According to them, all situations provoking MS are characterized by a condition in which the sensed vertical, as determined on the basis of integrated information from the visual system, the vestibular system, and the non-vestibular proprioceptors, is at variance with the subjective vertical that is expected from previous motion experience [7]. An alternative theory of MS has been proposed by Riccio and Stoffregen [8] based on relations between perception and the control of action (postural instability theory). The postural instability theory asserts that MS will be preceded and predicted by instabilities in postural control. Stoffregen and Smart [9] suggested that instability might occur when posture is controlled in the presence of imposed oscillations in the frequency range of spontaneous sway through a form of wave interference. When two systems oscillate at similar frequencies, the interaction of the waveforms can lead to instabilities (waveforms similar but out of phase). This theory predicts an increased incidence of sickness when external motion is imposed at a frequency range between 0.1 and 0.3 Hz, because this interferes with the naturally occurring sway activity [10]. Accordingly, MS has been found almost exclusively when imposed periodic motion includes frequencies from 0.08 to 0.4 Hz [11]. Vibration in this frequency range is characteristic of nauseogenic vehicles [11–14]. Optical motion at these frequencies is also sufficient to induce MS [9, 15–17].

Many studies have reported correlations between postural instability and MS [9, 17–22]. Postural instability has also been reported as an aftereffect of exposure to virtual environment systems [23–27]. However, according to Stoffregen and Riccio’s [28] theory, postural instability causes MS, instead of being caused by it. In order to test this theory, motion sickness and postural stability were assessed on standing participants exposed to a moving room that provided an optical simulation of body sway [9, 17, 29] or on seated participants exposed to a fixed-base flight simulator [30]. The results of these experiments showed that the sick participants exhibited more postural instability and that it preceded MS. Moreover, widening the distance between the feet, known to decrease the magnitude of sway, influenced motion sickness incidence [31]. However, some studies revealed the existence of subjects with unstable posture who never get sick and other studies have shown that MS may occur in subjects with stable posture, (for a review, see [32]). This apparent contradictory results regarding the relationship between MS propensity and postural instability may come from the absence of a clear definition for postural instability in terms of posturographic variables. Even though an increase in the spatial magnitude of body sway is usually interpreted as a decrease in body stability, there is no general agreement on the definition of stability based on the dynamic characteristics of postural sway. Moreover, the dynamic postural variables, like those extracted from the frequency spectrum of the body sway or from the long-range correlations in the dynamics of sway [33], are not necessarily a measure of postural stability but may reveal the underlying neural or sensorimotor processes which successfully stabilize the body.

The MS dependence on the low frequency range of postural oscillation, particularly around 0.2 Hz [6, 15, 34–37], may also arise from the misinterpretation of own-body motion. It has been shown that imposed accelerations above 0.2 Hz are usually perceived as translation of self through space, whereas imposed accelerations below that value are usually perceived as a change in direction of the gravitoinertial acceleration vector, i.e., tilt of self with respect to the assumed gravity vertical [38]. The region around 0.2 Hz would then be a crossover between these two perceptual interpretations and, thus, a frequency region of maximal ambiguity concerning the correct frame of reference for spatial orientation [39]. Duh et al. [16] elicited simulator sickness on their subjects when imposing concurrent oscillations of the body and the visual scene at slightly different frequencies. The amount of sickness was higher when the oscillation frequencies were around 0.06 Hz than around 0.2 Hz. However, simulator sickness peaks between 0.2 and 0.4 Hz with a reducing effect at lower and higher frequencies when the symptoms are purely visually induced [15].

Individual differences in MS susceptibility are large. A variety of predictors of MS has been examined over the years, like gender, age, experience, etc. The relationship between postural control as a predictor and MS susceptibility has not yet been clearly determined. Some studies showed that subjects, who are more likely to sway in the absence of motion stimulation (e.g. spontaneous sway), have high MS susceptibility during and after imposed or virtual motion [9, 21, 30, 40–43]. However, this relationship has not been found in other studies [17, 29, 44]. This lack of agreement in the results of these studies may have different origins, which will be discussed below and put in perspective as regards the experiments presented in this paper.

First, the contradiction may be due to the stimulus used to provoke MS. For example, with visually induced motion sickness (VIMS) the motion is implied and not real. While the visual system is necessary in VIMS, is is not necessarily required for inertial motion sickness, as is illustrated by the observation that blind people can become motion sick in vehicles [45]. For this reason, we did two experiments in our study, one involving the MS history for inertial motion (Exp. 1) and another involving MS symptoms triggered by visual motion (Exp. 2).

The contradictions may also be related to the dichotomous classification of subjects into well and sick groups. It can be suggested that the level of MS of sick participants may differ among these studies. In order to overcome these drawbacks, we used in the present study a continuous measure of MS, instead of separating the populations into well and sick groups.

Finally, direct comparisons among studies are difficult because different techniques have been used for measuring body motion and characterizing postural sway. As pointed out by Pavol [46], an important consideration in much clinical and basic scientific studies is which measures are best for detecting differences in postural sway. In the present study, selection of stabilometric parameters was made according to the recommendation of Schubert et al. [47], based on exploratory factor analysis. Moreover, it is recognized in the literature that fine aspects of the Power Spectral Density (PSD) profile of the postural sway signal may represent the normal variations in the basic mechanism for standing upright [10]. Hence, this study also concerns the relationship between propensity to MS and energy distribution in the sway signal spectrum. According to the wave interference theory, subjects whose sway spectrum have strong component around 0.2 Hz would be more prone to MS, since the symptoms of MS are more severe for motion frequency around this value. Testing hypotheses like this one can only be done inside a framework where the level of MS can be inferred from the shape of the Power Spectral Density (PSD) profile of spontaneous sway during stationary, upright stance. Establishing and testing this framework is the main goal of the present study.

## Materials and Methods

### Participants

43 participants participated in Exp. 1 (aged 20 to 43, mean = 25.8, 15 males) and 24 in Exp. 2 (aged 30 to 42, mean = 35.4, 24 males). All participants had normal or corrected to normal vision and reported no history of balance or neuromuscular disorder. They were all naïve to the aim of the study and gave their informed and written consent prior to their inclusion in the study. They were free to withdraw from the experiment at any time. The research was approved by the CPP sudEs5 local ethics committee from Centre Hospitalier Universitaire de Grenoble, requirement number CPP08-CRSS-01, and performed in accordance with the ethical standards specified by the 1964 Declaration of Helsinki.

### Experimental setup

#### Static posturography

A force-sensitive platform equipped with four strain gauges linked to a computer was used to record the displacements of the center of feet pressure (CoP) in the horizontal plane. The force-platform was developed in the French Armed Forces Biomedical Research Institute. It is made of two parallel steel plates (50×50×3 cm). The upper plate lays on four strain gauges (AG50C3SH10ef SCAIME) mounted near the corners of the lower plate, distant from each other by 40 cm. With regard to the position of the CoP, the mean precision for a 70 kg load applied on the center of the platform is inferior to 0.1 mm. The sampling frequency of the platform was 100 Hz. The CoP signal was filtered by a second-order low-pass filter with cutoff frequency at 10 Hz. The antero-posterior (AP) and medio-lateral (ML) axes are defined as being parallel to the horizontal borders of the platform. During data collection, the participant was instructed to align his/her straightforward direction along the positive AP direction of the force platform. The distance from ground to the upper surface of the device was 18 cm. Barefoot participants were asked to stand as still as possible on the force platform with feet close together and both arms relaxed on the side of the body with eyes open or closed. During the measurement with eyes open, they were asked to fixate to a vertical line located 1.5 m in front of them. Each trial was preceded by 10 sec of static posture for familiarization with the task, followed by 10 sec of rest. Once participants adopted the proper position and had stabilized their postural behavior, recording was started for a total duration of 30 sec.

#### Visually induced motion sickness (Exp. 2)

A large motor-driven half sphere (60 cm of diameter) which can be rotated around the observer’s line of sight was mounted 25 cm in front of the participant’s eyes. This covers a large portion of the participant’s vision field of view (around 100deg of visual angle). The inner surface of the sphere was painted white with a gray central circle. It was randomly covered by colored dots of various sizes (30% of surface). It was rotated clockwise at a constant velocity of 30deg/sec. Stimulation was performed with participants in upright stance. They were instructed to stare at the gray center during the stimulus presentation without following the dots with their eyes.

### Experimental procedures

#### Experiment 1

Motion sickness susceptibilities were rated on the responses to a standardized questionnaire (Motion Sickness Susceptibility Questionnaire, MSSQ [48]). The MSSQ is defined as the sum of two subscores, the MSA (MS susceptibility before 12 years old) and the MSB (MS susceptibility during the last 10 years). For younger subjects of our cohort (13 out of 43), the periods corresponding to the MSA and the MSB scores overlap. This could artificially increase the MSSQ score for those subjects [49] and, therefore, only the MSB was used in our analysis. Static posture was then measured, with eyes open and closed (with the order randomized across participants). There was a 30 sec rest break between the two trials.

#### Experiment 2

Participants only underwent the visual motion stimulation if they had no significant, spontaneous autonomic symptoms when questioned before the session. Static posture was measured at first with eyes open. After a 30 sec rest break, the visually induced motion test was started by presenting the stationary sphere for 10 sec followed by the movement of the sphere for 120 sec and the stationary sphere again for 10 sec. At the end of the test, the Simulator Sickness Questionnaire (SSQ [50]) was administered to assess how participants felt after exposure to the visual movement.

### Data Analysis

#### Traditional stabilometric parameters

For each postural trial, seven parameters were calculated from the CoP along the AP and ML directions: Length (total distance traveled by the CoP over the trial period, in mm), Area of the 95% confidence ellipse (in mm^2^), Alignment (displacement of the main direction of the CoP trajectory away from the AP axis, in deg), AP and ML range (differences between the two extreme position values of the CoP in the respective direction, in mm), AP and ML mean power frequency (MPF, in Hz). This set of parameters was chosen following the recommendations of Schubert et al. [47] and are expected to capture the relevant kinematic information available in the CoP signals. In Exp. 1, the parameters were computed for both open- and closed-eyes conditions, what resulted in a set of 14 stabilometric parameters. Exp. 2 had only the open-eyes condition, which yields a set of seven parameters. In order to assess how well these stabilometric parameters can predict the MS score (MSB for Exp. 1 and SSQ for Exp. 2), we used a linear model, as provided by the R statistical software [51]. In this model, the MS score was taken as the dependent variable and the stabilometric parameters were taken as the continuous independent variables. The irrelevant variables were eliminated from the model by using a stepwise model selection based on the Akaike Information Criterion [52]. Significance of the remaining terms in the linear model were assessed using regular F-tests.

#### Principal component analysis on power spectral density

The MPF, one of the stabilometric parameters computed from the CoP signal, can roughly indicate whether the sway of a participant is more concentrated in low or in high frequencies. In order to assess how the MSB and the SSQ scores are related to fine details of the sway spectrum, and hence get an insight into the role of the different sensorial systems influencing the propensity to MS, a principal component analysis (PCA) of the power spectrum density (PSD) was undertaken. We did separated analysis for both directions (AP and ML) of the CoP. A multi-taper estimation of the PSD was performed for the signals in each direction for the whole duration of the 30-seconds trial. For this purpose, we employed the psdcore function of the R statistical program [53], the number of tapers for each frequency being set to 4, what gives a good trade-off between frequency resolution and spectral uncertainty. The resulting PSD, originally specified at frequencies multiple of 1/30 Hz, was interpolated on a logarithmic frequency scale going from 1/30 to 10 Hz, yielding a 300-points representation of the spectrum. This logarithmic transformation of the frequency scale allows the description of finer details in the low frequency range, which would be masked if a linear scale was used. The amplitude in dB of the PSD for each trial can then be represented as a point in a 300-dimensional space. For Exp. 2, a PCA was applied on this space across the population. For Exp. 1, an extended 600-dimensional space was considered, which jointly represented the PSD computed for both open- eyes and closed-eyes conditions. This joint representation allows to parcel out the intra-participant similarities between the two experimental conditions.

The PC analysis yields a set of orthogonal directions in the space of PSD amplitudes, whose origin is the population mean spectrum. This population mean can be represented as a PSD curve, while each orthogonal direction defines a specific variation in the spectrum around the mean, what is usually called PC loading. Each PSD obtained in each trial of our experiments can then be projected along the PC directions. The projections on the PC directions are called the PC scores, which are normalized in terms of standard deviations (SD). The spectral shape of each PC can then be visualized by adding the population mean to the corresponding PC loading. It must be noted that the variables used as the PSD data space of the PCA are amplitudes sampled at successive frequency values, which cannot be assumed to be independent. Thus, our results are similar to PCA applied to time series [54], in the sense that the PC loadings will look as relatively smooth variations around the mean PSD, with smoothness being higher for the lower order PCs. Correlation tests between the PC scores and the MS scores (either MSB or SSQ) obtained for each participant were finally carried out.

## Results

### Experiment 1

MSB scores ranged in our population from 0 to 24, median value was 4.5, first quartile was 2.1, and third quartile was 9.0. These values are close to those tabulated in the literature for the normal population, which are 3.7, 1.2 and 8.0, respectively [48]. The distribution of the MSB values of our cohort is not significantly different from the tabulated distribution (Kolmogorov-Smirnov test, *D* = 0.134, *p* > 0.42), which indicates that the sample was representative of the normal population. The mean MSB score was 6.5 (SD 6.11).

**Fig 1:**
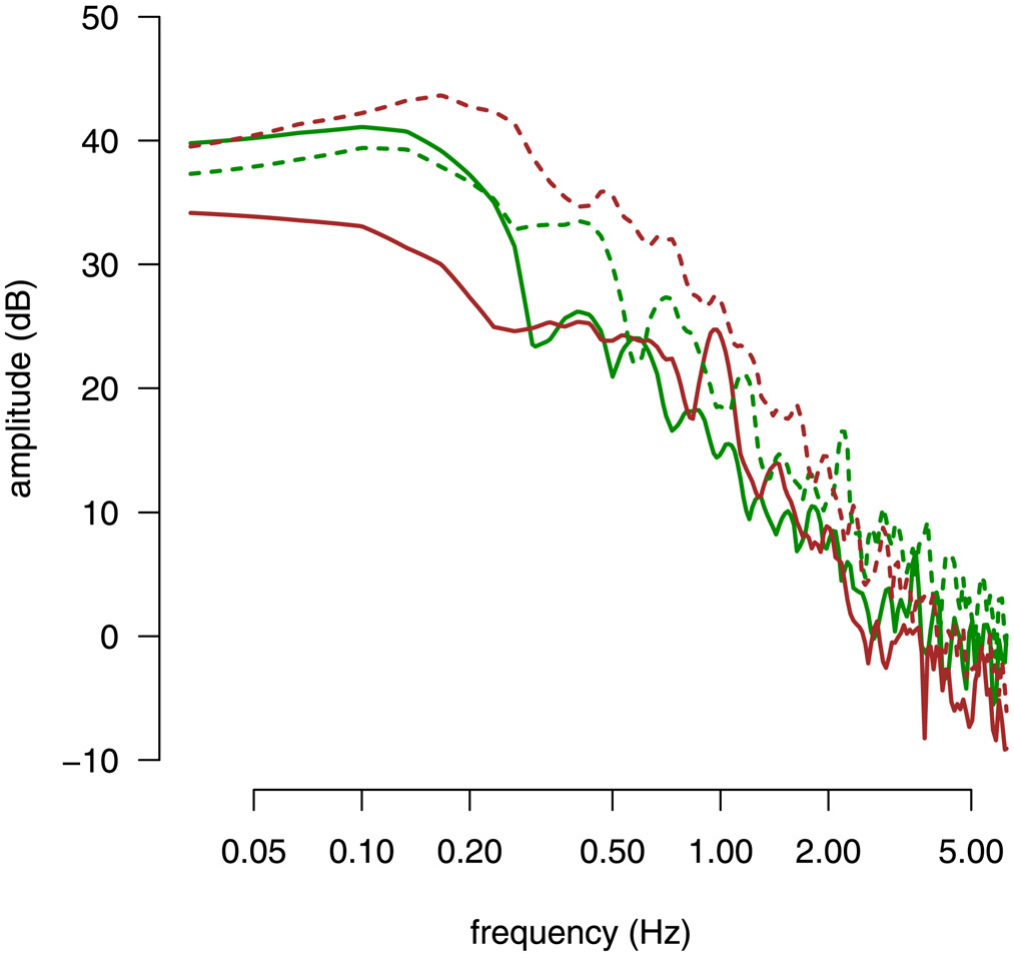
Power spectrum density curves for two participants representative of extreme cases in Exp. 1. The power spectrum of the CoP signal in the AP direction for two participants in Exp. 1 are shown. Each participant is indicated by a different color (brown or green). Solid and dashed lines indicate, respectively, the closed-eyes and the open-eyes conditions. The participants represented in this figure are indicated by colored circles (respectively in brown and green) in the bottom right panel of Fig 2.

The reduced linear model obtained by the stepwise procedure was the following: *MSB* = 11.5 − 0.0286*Length*_*O*_ + 0.205*Range*_*AP*/*C*_ + 22.6*MPF*_*AP*/*O*_ − 17.0*MPF*_*ML*/*C*_. In this formula, the subscripts with *O* and *C* in the variables relate to the open-eyes and closed-eyes conditions respectively. For the 1D variables, the direction (either AP or ML) are also indicated in the subscripts. Besides the coefficient for *MPF*_*ML*/*C*_., which is only marginally significant, all the others are significantly different from zero (*Length*_*O*_: *F*[1, 38] = 5.62, *p* < 0.05; *Range*_*AP*/*C*_: *F*[1, 38] = 5.20, *p* < 0.05; *MPF*_*AP*/*O*_: *F*[1, 38] = 7.15, *p* < 0.02). Overall, the higher the MSB score the larger the AP range when eyes were closed, the shorter the length and higher the AP MPF when eyes where open. In Fig 1, PSD curves computed on the AP direction for two different participants in Exp. 1 are shown, in both open-eyes and closed-eyes conditions.

**Fig 2:**
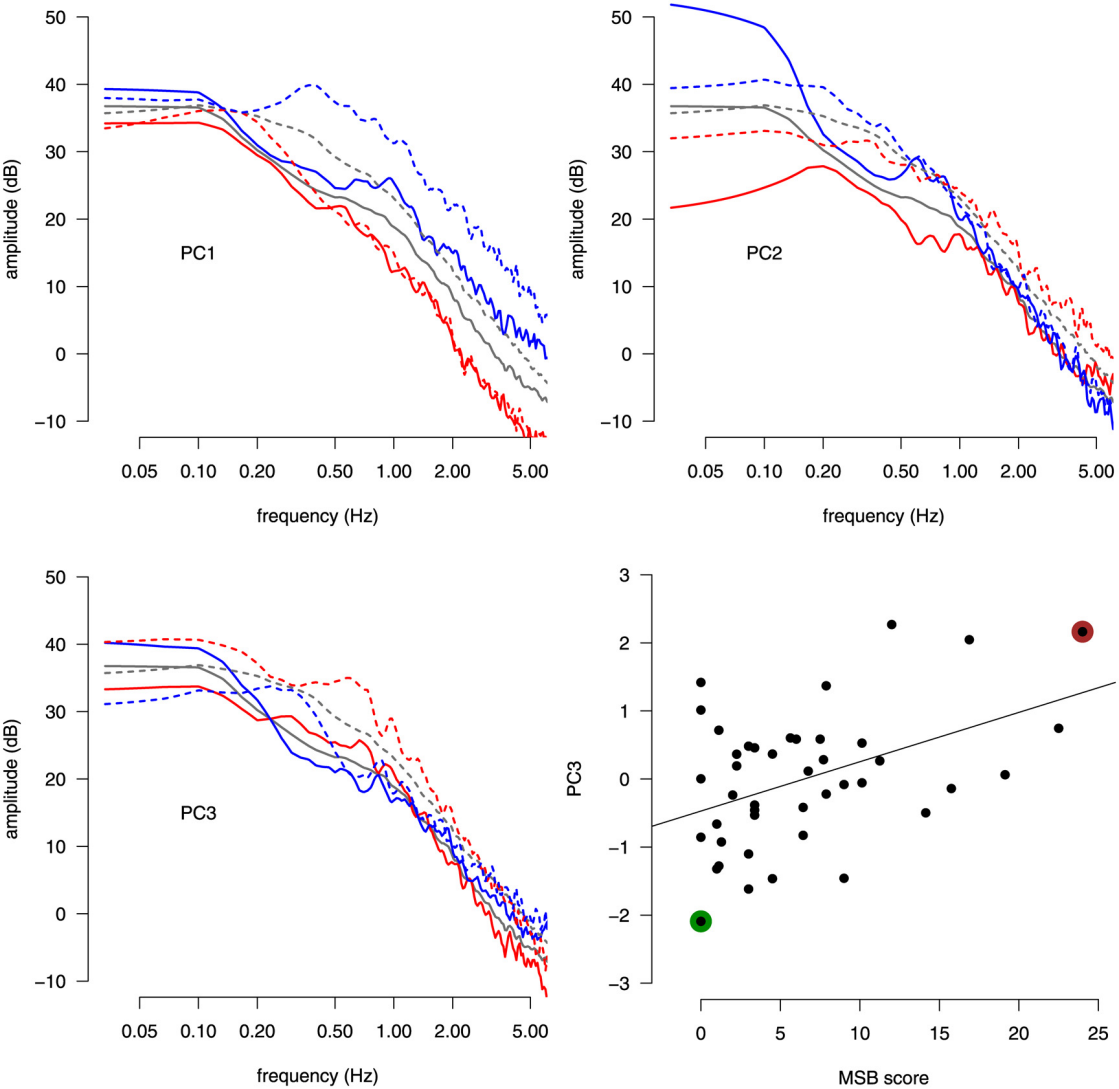
Principal component analysis of CoP data in the MSSQ experiment. The first three principal components of the power spectrum of the CoP signal in the AP direction are shown in the top panels and in the bottom left panel. The cohort average spectrum is shown with gray lines. For each PC, the associated PC loading variation around the average spectrum are represented with red and blue lines, which correspond to PC scores of +3 SD and -3 SD, respectively, along the PC direction. The open-eyes and closed-eyes conditions are shown with solid and dashed lines, respectively. The scatter plot in the bottom right panel shows the MSB score against the value of PC3 in the cohort (each participant is represented by a dot). The two colored dots indicate the participants whose PSD curves are represented in Fig 1. The solid line represents the linear regression fit between the MSB score and the value of PC3.

The loadings of the first three PCs obtained from the PCA applied to the AP PSD data are shown in Fig 2. These three components account for 60.9% of the total variance (28.9%, 23.5%, 8.5%, respectively). The mean spectrum for the open-eyes condition (solid gray line in Fig 2) differ from the mean spectrum for the closed-eyes condition (dashed gray line in Fig 2). In the closed-eyes condition, more energy is allocated in the frequency region above 0.1 Hz.

Each PC has a natural explanation. PC1 accounts for the tendency of a participant to use more or less energy uniformly across the entire frequency spectrum. PC2 represents the tendency to allocate either more or less energy in the range of frequencies below 0.5 Hz. This component correlates well with the MPF computed in the AP direction (open eyes: *R* = 0.845, *t*[41] = 10.2, *p* < 0.001; closed eyes: *R* = 0.403, *t*[41] = 2.82, *p* < 0.01). Variations along the PC3 axis imply an opposite allocation of energy below and above 0.25 Hz, in its open-eyes part. In its closed-eyes part, PC3 induces no variation for frequencies around 0.3 Hz, while the spectrum either increases or decreases for any other frequencies. It should also be noted that PC3 has a reversal effect in low frequencies for the closed-eyes and open-eyes parts. The only component that correlated with the MSB score was PC3 (*R* = 0.443, *t*[41] = 3.16, *p* < 0.01). Along the PC3 in the open-eyes condition, participants with high MSB score use less energy in the low frequency range (below 0.25 Hz) than participants with low MSB score. The opposite pattern was observed for frequencies in the range 0.25–1.0 Hz. However, in the closed-eyes condition, participants with high MSB score use more energy across the entire frequency spectrum. The opposite pattern is associated for participants with low MSB scores. The PCs for the ML direction showed a similar pattern than those for the AP direction, but no component was correlated with the MSB score, so they are not reported in this article.

In order to check whether the obtained results would depend on the particular choice of the number of tapers of the PSD algorithm, we ran a sensitivity analysis by computing the PSD with number of tapers equal to 2 and 8. These values, which correspond to half and double of the value of 4 tapers used in this study, yielded noisier or smoother PSD curves, respectively. Essentially, the same results were obtained, with the correlation between the MSB score and PC3 remaining significant for the number of taps 2 and 8. Also, the shape and the variances of the PCs were similar in all three cases.

### Experiment 2

**Fig 3:**
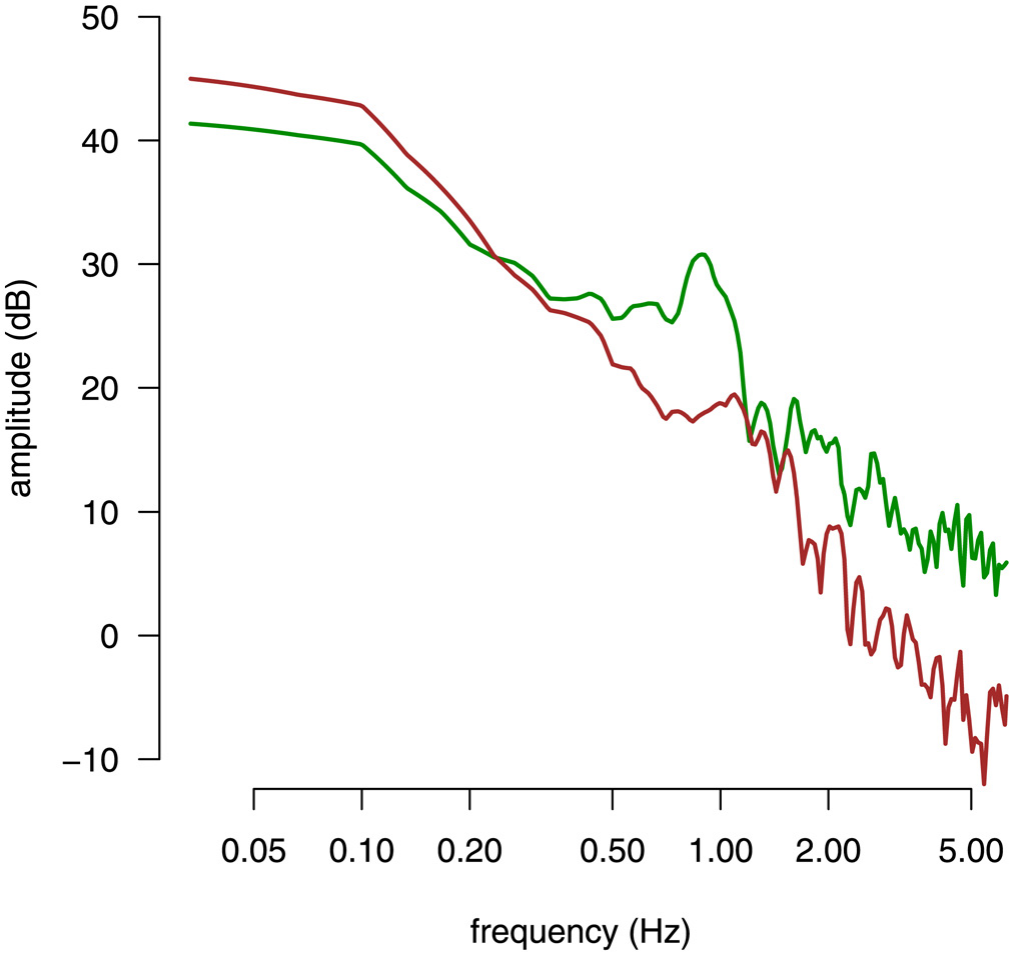
Power spectrum density curves for two participants representative of extreme cases in Exp. 2. The power spectrum of the CoP signal in the AP direction for two participants in Exp. 2 are shown. Each participant is indicated by a different color (brown or green). The participants represented in this figure are indicated by colored circles (respectively in brown and green) in the bottom right panels of Fig 4.

The Total Severity score, inferred from the SSQ, has been used as a general indicator of MS induced by the visual motion device [55]. The mean score was 28.7 (SD 32.1). The reduced linear model obtained by the stepwise procedure was the following: *SSQ* = 67.3 − 163*MPF*_*AP*_. The coefficient for *MPF*_*AP*_ is significantly different from zero (*F*[1, 23] = 6.23, *p* < 0.05). The higher the SSQ score, the lower the AP MPF. In Fig 3, PSD curves computed on the AP direction for two different participants in Exp. 2 are shown.

**Fig 4:**
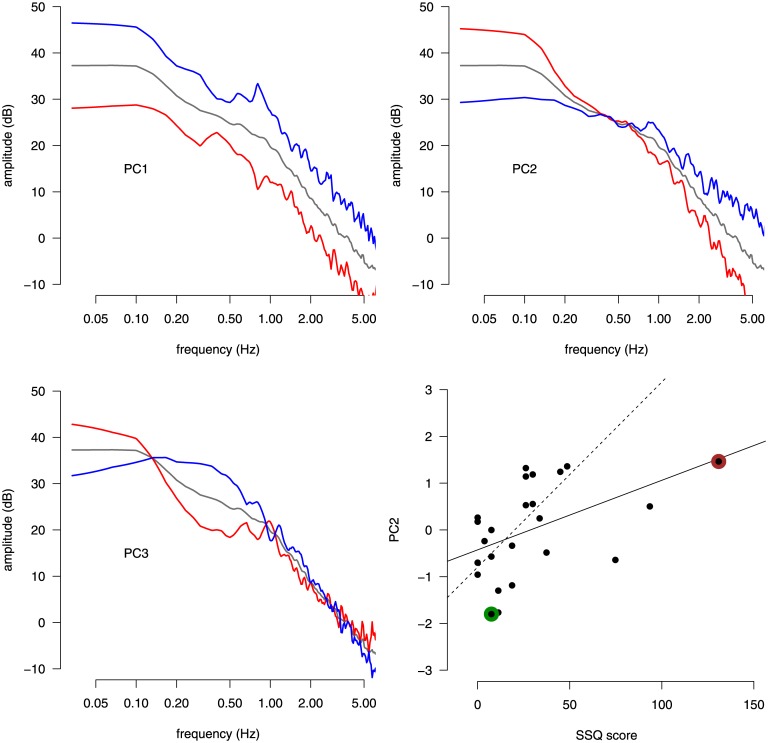
Principal component analysis of CoP data in the SSQ experiment. The first three principal components of the power spectrum of the CoP signal in the AP direction are shown in the top panels and in the bottom left panel. The cohort average spectrum is shown with gray lines. For each PC, the associated PC loading variation around the average spectrum are represented with red and blue lines, which correspond to PC scores of +3 SD and −3 SD, respectively, along the PC direction. The scatter plot in the bottom right panel shows the SSQ score against the value of PC2 in the cohort (each subject is represented by a dot). The solid line represents the linear regression fit between the SSQ score and the value of PC2. The two colored dots indicate the participants whose PSD curves are represented in Fig 3. The dashed line represents the linear regression fit when the three subjects with the highest values of the SSQ score are dropped.

The loadings of the first three PCs obtained doing the PCA on the AP PSD data are shown in Fig 4. These three components account for 76.0% of the total variance (38.2%, 26.3%, 11.4%, respectively). As in Exp. 1, each PC here has a natural explanation. PC1 accounts for the tendency of a subject to use more or less energy uniformly across the entire frequency spectrum. PC2 represents the balance between low and high frequencies, with a pivoting frequency around 0.3 Hz. This component correlates well with the MPF computed in the AP direction (*R* = −0.849, *t*[22] = −7.53, *p* < 0.001). PC3 indicates the opposite allocation of energy between two regions: 0–0 2 Hz and 0.2–1.0 Hz. The only component that correlated with the SSQ score was PC2 (*R* = 0.477, *t*[22] = 2.544, *p* < 0.02). MS participants have more energy in the low frequency range. The three participants with the higher values of the SSQ score seem to influence the correlation between PC2 and SSQ. In order to rule out this possibility, we computed the correlation when removing these three participants and it remains significant (*R* = 0.604, *t*[19] = 3.30, *p* < 0.01). Similarly to what we observed in Exp. 1, the PCs for the ML direction showed a similar pattern to those for the AP direction, but no component was correlated with the SSQ score, so they are not reported in this article.

In order to check whether the obtained results would depend on the particular choice of the number of tapers of the PSD algorithm, we ran the same sensitivity analysis as described in the Results section of Exp. 1 above. Essentially, the same results were obtained with the correlation between the SSQ score and PC2 remaining significant for the number of tapers 2 and 8. Also, the shape and the variances of the PCs were similar in all three cases.

## Discussion

The present investigation addressed the potential associations between naturally occurring sway activity and motion sickness susceptibility. Although classical measures of stabilometry (i.e. CoP lengh, range, etc.) were somehow related to MS sensitivity in our experiments, postural responses in the frequency domain showed interesting differences in spontaneous sway between persons who are susceptible to MS and those who are not. Furthermore, different patterns of power spectrum distribution were associated with MS related to inertial motion (Exp. 1) and MS provoked by exposure to visual motion (Exp. 2).

### Sickness induced by inertial motion

Concerning MS susceptibility, the postural sway in the absence of any imposed body motion showed different patterns between participants who are susceptible and non-susceptible to MS. Whereas MS can be triggered without visual input, as illustrated by the observation that blind people can get motion sick [45], our main result concerns MS and postural sway when eyes were open. As shown in Experiment 1, subjects with higher MSB scores presented higher values of AP MPF with eyes open. MPF is a frequency-weighted average of the power spectrum. Therefore, higher MPF values represent stronger contribution of the high frequency components of the sway signal. Differences were also observed in the distribution of energy along the power spectrum of the sway signal. For AP oscillations in the open-eyes condition, two trends were observed in the 3rd PC, which correlates with MS propensity. In comparison with participants with no MS, participants with MS have less energy in the lower frequency range (under 0.25 Hz), and more energy for frequencies between 0.25 and 1.0 Hz. These results did not suggest a strong component of postural oscillation around 0.2 Hz for MS participants, as hypothesized in the Introduction. However, these differences in the spectral characteristics of the CoP signal may be related to differences in the relative contribution of vestibular, visual and somatosensory systems for the maintenance of upright stance under unperturbed sensory conditions. It was originally suggested that within the neural pathway for postural stabilization, each of these three systems has a separate frequency range for optimal operation [56–59]. It is assumed that low frequencies account for visuovestibular regulation (0–0.5 Hz), medium frequencies for cerebellar participation (0.5–2 Hz), and high frequencies for proprioceptive participation (>2 Hz). Thus, these results may suggest that participants who had a lower MS severity score used more visuovestibular postural regulation. However, since MS participants have a nearly flat spectrum in the 3rd PC, no conclusion may be drawn about the relative participation of these systems in their postural stance.

Regarding the difference in the frequency profile of the 3rd PC between the open-eyes and the closed-eyes conditions, the following remarks may be drawn. On the one hand, for participants with no MS, the spectral energy distribution of the 3rd PC for both conditions differed, with more energy being allocated in the low frequency range (below 0.2 Hz) in the open-eyes condition. On the other hand, the 3rd PC of MS participants presented more energy across all frequencies in the closed-eyes condition in comparison with the open-eyes condition. This last result suggests that MS participants do not re-weight sensory information for maintaining stability according to the sensory context (presence or absence of vision).

### Visually induced motion sickness

Circular vection produced a high score of motion sickness in 50% of the participants (SSQ scores >20) within a short interval of time. Postural stability measures registered before the onset of visual roll movement were correlated with self-reported symptoms of SSQ following stimulation. On the one hand, the higher the SSQ score, the lower the AP MPF. Thus MS participants showed more postural sway in the low frequency range. On the other hand, differences were also observed in the 2nd PC of the power spectrum density for the AP direction. For low frequency, particularly under 0.15 Hz, energy distribution was higher for motion sick participants. There are claims that vision contributes to postural stabilization in the low frequency range of body movements. Vision stabilizes sway primarily at frequencies below 0.1 Hz [60, 61]. Thus, these results may suggest a higher visual participation in postural stance for participants who tend to be motion sick. Spontaneous oscillations of the body cause slip of the image on the retina that is subsequently used to stabilize the body. The precision of visual stabilization of posture depends on how effectively the information on body oscillations can be extracted from the visual flow. It can be suggested that, during vection, the low frequency of the visual stimulation would interfere with the naturally occurring sway activity, inducing motion sickness [10]. The differences observed in posture between MS and non-MS participants could also be related to the selection of the spatial frame of reference used in postural control [62, 63]. Thus, it could be suggested that postural control of MS participants in Exp. 2 would have a higher dependence on the visual input, which could enhance the visuo-vestibular conflict induced by the optokinetic stimuli.

### Different spectral characteristics of postural sway according to the provocative environments

Our results suggest that sensory weighting involved in postural stabilization is related to MS sensitivity. However, as shown in the previous sections, different results were observed for inertial (Exp. 1) and visually-induced (Exp. 2) motion. This differences in the results between both experiments, could be related, in principle, to the fact that both cohorts were not necessarily matched for age and anthropometric measures. However, further analysis showed that, for the open eyes conditions, the relevant postural parameters (namely *Length*_*O*_ and *MPF*_*AP*/*O*_) did not differ significantly between the two cohorts. Furthermore, PCA applied on the combined cohort did not reveal differences in the spectral variation between both cohorts (see S1 Appendix for details). The different relationship between MS and postural sway in both experiments can be related to the questionnaire used. In Exp. 1, the original intent was to measure general motion susceptibility. However, in the MSSQ, sickness is defined as feeling queasy or nauseated or actually vomiting. The SSQ used in Exp. 2 rates nausea but also visuomotor and disorientation symptoms. In our study, the disorientation was the most important symptom factor, followed by the visuomotor subscale, and nausea was the weakest sickness contributor. This result is not surprising as symptoms provoked by motion of the visual field are less severe (e.g. vomiting is rarely reported) than the sickness that occurs with inertial motion [1]. Thus, while MS was defined as nausea in Exp. 1, it was not the case in Exp. 2. Using a single questionnaire (for example, the misery scale, MISC, [64, 65] would allow the MS susceptibility to be defined on a unique scale in both experiments. However, it must also be taken into account that the specific MS symptoms may depend on the nature of the provocative stimulus [66]: simulator sickness typically involve visually-induced motion stimuli, as opposed to traditional forms of MS that are caused by inertial motion.

### Postural sensory weighting and motion sickness

The present experiment supports the hypothesis that the characteristics of postural control in the absence of motion would be correlated with susceptibility to motion sickness. Thus, the level of MS can be predicted from the shape of the Power Spectral Density profile of spontaneous sway. The results of the present experiment do not contradict the postural instability theory of MS, which argues that the degree of the disruptive effect on postural control is related to the similarity between the ranges of oscillation of imposed and natural motion [8]. However, the analysis of the sway frequency spectrum provided insight into the intersubject differences in the use of postural control subsystems and how these differences are related to MS susceptibility. Contrary to the postural instability theory, we propose that a sensory weighting mechanism could explain both the large individual differences in MS sensitivity and the fact that MS level may vary according to the MS stimulus (real *vs* visually-induced motion).

Our results also suggest that MS participants do not re-weight sensory information for maintaining stability according to the sensory context (presence or absence of vision in Exp. 1). A generally held view of the postural control system is that multiple sensory inputs are dynamically re-weighted to maintain upright stance as sensory conditions change [60, 67]. Attributing differences in MS to sensory reweighting alone is here speculative, the employed experimental procedure did not permit a systematic examination of sensory-reweighting during motion stimulation. However, in line with this hypothesis, Nachum et al. [68] observed that individuals susceptible to *mal de débarquement* have reduced reliance on vestibular and visual inputs and increased dependence on the somatosensory system for the maintenance of balance.

Accurate organization of sensory information is critical for maintaining balance within the variety of environments encountered in daily life. The ability to re-weigh sensory information may be important for maintaining stability when an individual goes from one sensory context to another. More experiments must be done in order to understand if and how subjects adapt the weighting of visual, vestibular, and somatosensory inputs in postural control, when placed in the different moving environments.

## Supporting Information

S1 Appendix Comparison between cohorts of Exp. 1 and Exp. 2. Detailed analysis showing that there are no significant differences between the cohorts of Exp. 1 and Exp. 2 as regards the relevant traditional stabilometric parameters and as regards the variations in the PSD profiles in the AP direction (open-eyes condition) as obtained by PCA.
(PDF)
