# Supplementary material for: Relationship between Spectral Characteristics of Spontaneous Postural Sway and Motion Sickness Susceptibility
Source: PLoS One. 2015 Dec 14;10(12):e0144466. doi: 10.1371/journal.pone.0144466 (PMC4685995; doi:10.1371/journal.pone.0144466)
Supplement: S1 Appendix — Detailed analysis showing that there are no significant differences between the cohorts of Exp. 1 and Exp. 2 as regards the relevant traditional stabilometric parameters and as regards the variations in the PSD profiles in the AP direction (open-eyes condition) as obtained by PCA. (PDF) [file pone.0144466.s001.pdf]

S1 Appendix. Comparison between the cohorts in Exp. 1 and Exp. 2

Supporting Information for the article

"Relationship between spectral characteristics of spontaneous postural sway and motion sickness susceptibility"

by R. Laboissière, J.-C. Letievant, E. Ionescu, P.-A. Barraud, M. Mazzuca, and C. Cian.

Introduction

In the study presented in the paper, differences were found between the results of Exp 1 and Exp. 2, as regards the correlation between postural sway parameters with the scores obtained from the Motion Sickness Susceptibility (MSSQ) and the Simulator Sickness (SSQ) Questionnaires, respectively.

It has been shown in previosu studies that stabilometric parameters are affected by anthropometric parameters (**??**) and by age (**??**). A legitimate issue that may be raised regarding our study is whether the differences in our two cohorts could explain the differences in the results from both experiments. Indeed, the cohorts of Exps 1 and 2 were not matched for age, weight, and sex (height of the participants was not recorded). In the present document, we report additional tests to assess the differences in the postural sway parameters between both cohorts.

Traditional stabilometric parameters

T-tests were run for each one of the seven traditional stabilometric parameters used in the study. We only used the parameters computed for the open-eyes condition, since there was no closed-eye condition in Exp. 2. Since the size differ for both cohorts, as well as the within-group variances, the Satterthwaite approximation to the degrees of freedom was used. The results are shown in Fig. S1–**??**.

In our study, we found the following linear models relating the postural parameters to the sickness scores issued from the MSSQ (Motion Sickness Susceptibility Questionnaire) and the SSQ (Simulator Sickness Questionnaire):

$$MSB = 11.5 - 0.0286 Length_O + 0.205 Range_{AP/C} + 22.6 MPF_{AP/O} - 17.0 MPF_{ML/C}.$$
$$SSQ = 67.3 - 163 MPF_{AP}$$

The only parameters related to the open-eyes condition in the models above are $Length$ and MPF_{AP}, which do not show significant differences between the cohorts of both experiments. Thus, we could rule out any differences in the interpretation of the above models that comes from the mismatched cohorts.

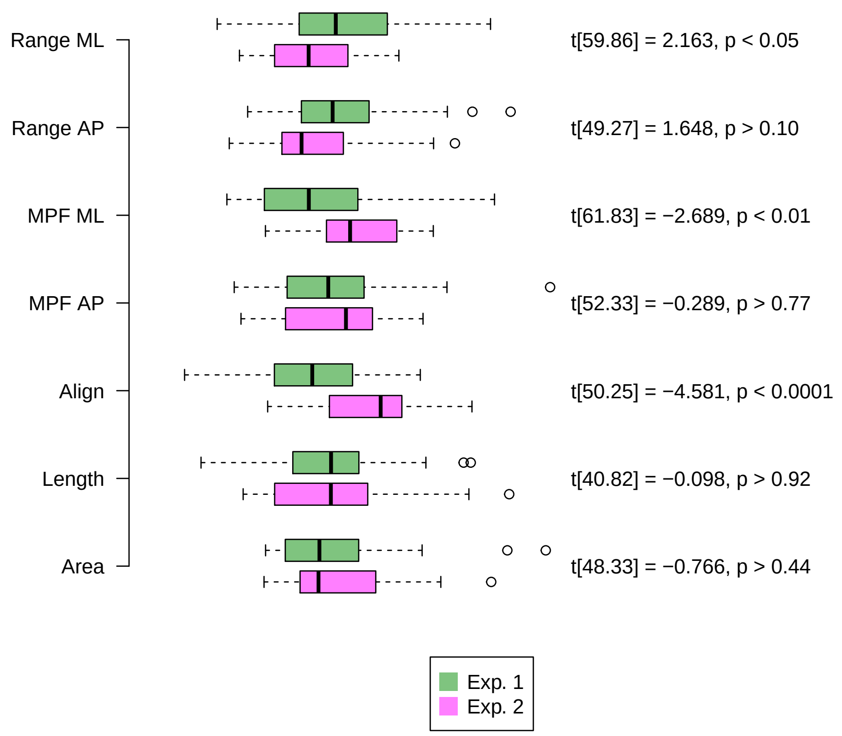

Figure S1 – 1 – Comparison of the traditional stabilometric parameters between the cohorts of Exp. 1 and Exp. 2. Box plots for each of the seven stabilometric parameters used in the study are shown, separately for Exp. 1 (green) and Exp. 2 (magenta). For display purposes, the standard deviation has been normalized and the means have been aligned across the parameters (and, hence, the horizontal scales have been suppressed). The results of the t-tests are shown at the right margin of the plot.

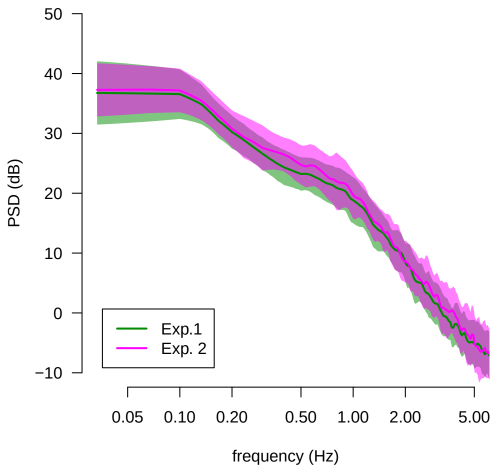

Figure S1 – 2 – Mean PSD profiles and standard deviation for both cohorts. The mean PSD profile, in the AP direction, for each cohort is show with solid lines (green for Exp. 1 and magenta for Exp. 2). The one standard deviation bands, are represented as colored strips around the mean profiles.

Power Spectrum Density

We also compared the power spectrum density (PSD) profiles for the cohorts in Exps. 1 and 2. As for the traditional-parameters stabilometric parameters, we only used the open-eyes condition. Furthermore, we present here only the results for the antero-posterior (AP) direction, since there was no significant result for the medio-lateral (ML) direction.

The comparison was done in two steps. First, we looked at the shape of the mean PSD profiles and its dispersion around the mean for each cohort. The result is shown in in Fig. S1–**??**. Notice that although there is a slight difference in the mean PSD profiles around 0.5 Hz, the one-standard variation regions largely overlap across the frequency range from 0 to 5 Hz. This means that both cohorts are similar in terms of gross spectral profiles.

Second, we assessed how much the cohorts would vary in the Principal Component Analysis (PCA) representation. For doing this assessment, we computed the PCA on the PSD profiles using the combined cohorts of 43 subjects of Exp. 1 together with the 24 subjects of the cohort of Exp. 2. The PC analysis was done for the whole set of 67 PSD profiles in the AP direction, using the technique described in section "Principal component analysis on power spectral density" of the paper. The six first PCs, which account for over 83% for the total variance, are shown in Fig. S1–**??**.

We then projected the original profiles for each of the 67 participants onto the axes of the rotated subspace spanned by the first six PCs obtained in the analysis. The result is shown in Fig S1–**??**, were the scatter plots are shown in $PC_i \times PC_j$ separate plots.

We tested for the difference in the mean values of the two cohorts by running a MANOVA

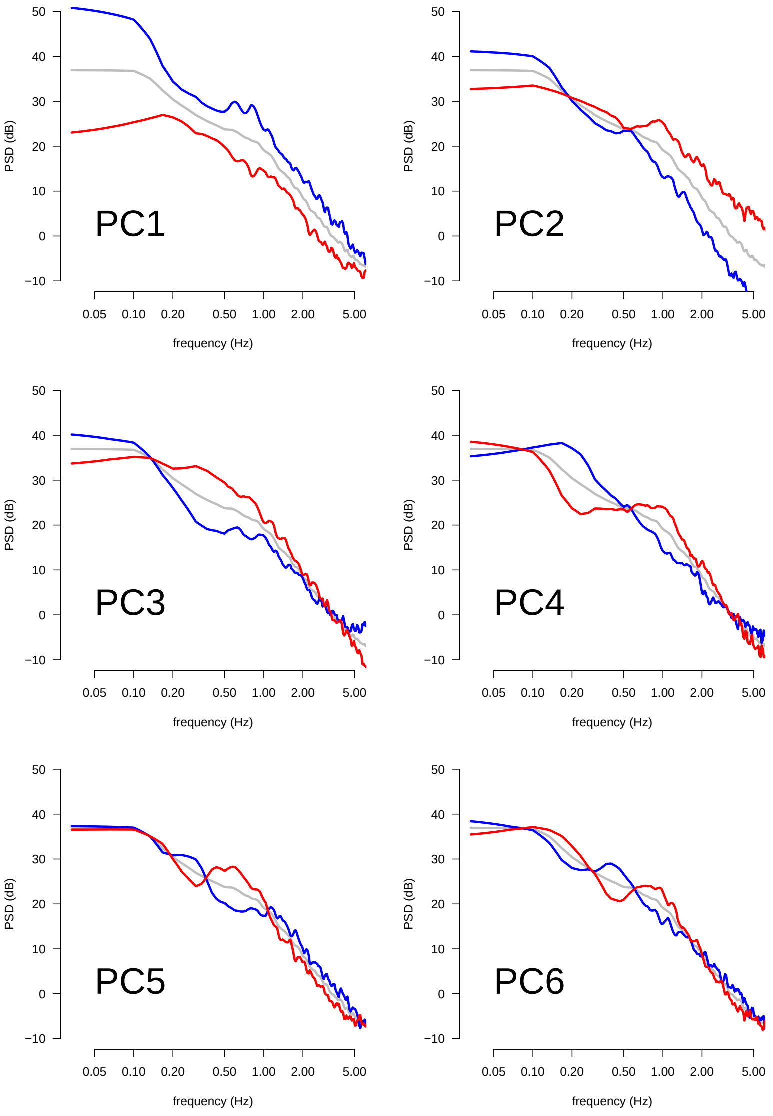

Figure S1 – 3 – First six principal components for the PCA applied to the PSD profiles of the open-eyes condition (AP direction) of the combined cohort. The mean PSD profile is shown with gray lines. The −3 SD and +3 SD lines are shown in blue and red lines, respectively.

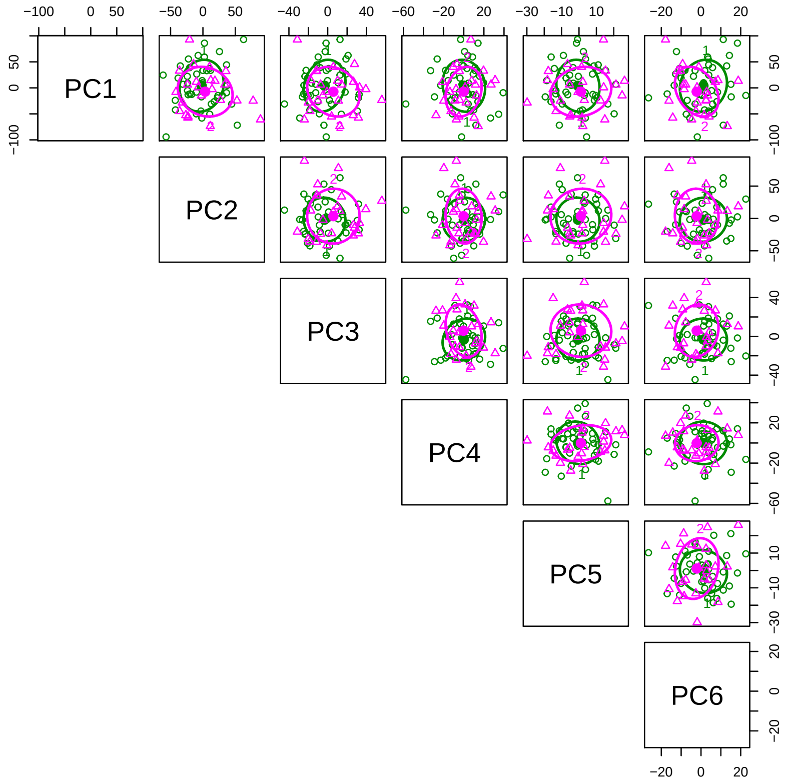

Figure S1 – 4 – Projection of the individual PSD profiles into the transformed space defined by the six first PCs. Scatter plots for each 2D subspace, formed by the 2 by 2 combination of PC1 through PC6, are show in the figure. Green circles and magenta triangles represent the cohorts of Exp. 1 and Exp. 2, respectively. The filled dots represent the mean values of each cohort and the curves stand for the 50% confidence ellipses.

in the 6D coordinate space (P1 through PC6), having the experimental cohorts (Exp. 1 vs. Exp. 2) as the independent variable of the analysis. The value of the Pillai's trace is 0.105, which is not significantly different from zero (approx. $F[6,60] = 1.173$, $p > 0.33$).

Similarly to what we concluded for the traditional postural parameters, we did not see significant differences in the PSD profiles for both cohorts, even though they are not matched in age and sex. Moreover, the PC analysis seem to indicate that, at least as regards the energy distribution along the sway spectrum, participants in both cohorts seem to be representative of a general population. This also increases the confidence in PCA of the PSD profiles as an appropriate method for dimensionality reduction in postural sway studies.
